# Premium IOLs in Glaucoma

**DOI:** 10.5005/jp-journals-10008-1138

**Published:** 2013-05-09

**Authors:** Parul Ichhpujani, Shibal Bhartiya, Anuj Sharma

**Affiliations:** Assistant Professor, Glaucoma Services, Department of Ophthalmology Government Medical College and Hospital, Chandigarh, India; Glaucoma Faculty, Department of Ophthalmology, Fortis Memorial, Research Institute, Gurgaon, Haryana, India; Junior Resident, Department of Ophthalmology Government Medical College and Hospital, Chandigarh, India

**Keywords:** Glaucoma, Intraocular implant, Multifocal IOL, Toric IOL.

## Abstract

Advanced technology or premium intraocular lenses have been developed to meet the patient expectations of perfect distance and near vision without the need for spectacles. Careful patient selection is critical when implanting these implants. This brief review focusses mainly on multifocal and toric IOLs and their application and limitations in patients with glaucoma.

**How to cite this article:** Ichhpujani P, Bhartiya S, Sharma A. Premium IOLs in Glaucoma. J Current Glau Prac 2013;7(2): 54-57.

## INTRODUCTION

All of us would have come across patients with glaucoma complaining of decreased vision. On visual acuity assessment, some have reduced vision while some may have excellent visual acuity, sometimes even 20/20.

Studies have shown that, although these patients may have good visual acuity, their complaints are genuine, as they often have decreased contrast sensitivity–an important visual function for day-to-day activities.^1,2^

Contrast sensitivity is the ability to detect differences between shades of light and dark and discern sharp edges. Evidence reveals that decreased contrast sensitivity is correlated with visual field loss in patients with glaucoma and the disease affects contrast sensitivity preferentially as compared with visual acuity.^1^

These days' patients with glaucoma, just like their nonglaucoma friends and folks, have growing expectations for a full range of vision and decreased dependence on glasses after cataract surgery.

### What Happens with the Aging of the Lens?

The decrease in visual acuity and contrast sensitivity that occurs with age is partially caused by changes in the lens related to increased wavefront aberration. In youth, the lens balances for the positive spherical corneal aberration by inducing negative spherical aberration; however, as the senility creeps over the lens, this decreases until the point when the lens itself also produces positive spherical aberration. It has been hypothesized that an IOL that would correct for corneal spherical aberration would increase contrast sensitivity in the pseudophakic eye.

### Is There a Solution?

Potential of an intraocular implant to affect contrast sensitivity, scotopic/mesopic vision, visual field testing, and structural imaging, as well as for anatomic features relevant to glaucoma patients, such as small pupils and capsular and zonular issues, to affect vision outcomes must be taken into account when choosing an IOL.

Great advancements have taken place in the field of cataract surgery and intraocular implants in the past few decades. Traditional IOLs are spherical and monofocal. The newer ‘premium IOLs'; aspheric, multifocal, accommodating and toric lenses offer an edge over the traditional ones.

There is currently a paucity of scientific literature as regards multifocal and newer accommodative IOLs in patients with concurrent ocular disease and patients with glaucoma.

### How Premium IOLs may help Contrast Sensitivity in a Glaucomatous Eye?

These lenses reduce spherical aberrations, and thus, decrease the glare, halos, and other optical phenomena that give rise to patients' complaints. Moreover, these IOLs have been shown to increase contrast sensitivity in patients in which they were implanted. This situation may be of special importance in patients with glaucoma, as they are already suffering from decreased contrast sensitivity.

*Aspheric IOLs*: A number of studies have shown that aspheric IOLs improve both mesopic and scotopic contrast sensitivity,^3-12^ whereas some studies have shown improvement only in contrast sensitivity under mesopic conditions,^13-15^ and still others have shown no improvement in contrast sensitivity,^16,17^ although some only evaluated patients under scotopic conditions.^18^

*Blue-filtering IOLs*: The impact of blue-filtering IOLs on contrast sensitivity has been variably reported to have shown no difference in contrast sensitivity,^19-21^ to a subjective increase in contrast perception.^22^ Another study revealed an increase in contrast sensitivity in patients with diabetes;^23^ while others have reported an improvement at the lower spatial frequencies^24^ and at the middle spatial frequencies.^25^

*Multifocal IOLs*: Multifocal IOLs (MFIOLs), on the other hand, cause a decrease in contrast sensitivity which is worse for near as compared to distance. The mesopic contrast sensitivity is worse than photopic, and the loss is greater at higher *vs* lower spatial frequencies following multifocal IOL implantation. This decrease in contrast sensitivity is considered to be more so with refractive than diffractive IOLs.

Patients implanted with the AcrySof ReSTOR^26,27^ (Alcon Laboratories, Fort Worth, Texas, USA) *vs* the AcrySof SA60AT (Alcon Laboratories) were reported to have a statistically lower monocular photopic contrast sensitivity. ReSTOR, ReZoom (Abbott Medical Optics, Santa Ana, California, USA) and Tecnis lenses all slightly decreased contrast sensitivity.^28^ The Array multifocal IOL (Abbott Medical Optics) has been associated with reduced contrast sensitivity at low contrast levels.^29,30^

All of these analyses were performed with the previous generation of spherical multifocal IOLs and with the advent of aspheric multifocal IOLs; some of the loss of contrast sensitivity may be mitigated.

Despite this, the use of multifocal IOLs in patients of glaucoma must be with extreme caution. These lenses are contraindicated in patients with moderate to severe disease, and extreme caution is advocated for patients of ocular hypertension, glaucoma suspects as well as mild disease.

Accomodative IOLs, on the other hand, will not induce a loss of contrast sensitivity, but the crystalens models have all suffered from "Z" syndrome (an aberrant folding of the IOL induced by capsular bag contraction) which seems more frequent in patients with pseudoexfoliation, and that might also be difficult to diagnose in eyes with small pupils.

### How Structural Alterations in Glaucoma can Influence Premium IOL Implantation?

Pseudoexfoliation is related to both glaucoma and cataract. Patients with pseudoexfoliation have a tendency to have a poor response to pharmacologic dilation and may have weakened zonules, which may manifest as iridodonesis, phacodonesis or lens subluxation/dislocation. These factors increase the risk of intraoperative zonular dialysis and postoperatively these patients may have an increased risk of not only posterior capsular opacification but also capsular phimosis and IOL dislocation.^31,32^ Patients with PXF may have higher pressures in the postoperative phase.^33^

Toric IOLs might also not be successful in patients with an unstable capsular bag, or pseudoexfoliation and/or weak zonules, as the lens and bag may rotate or tilt once implanted, altering the patients vision. There is a potential error if a toric IOL is implanted at the same time a glaucoma procedure is done, since a glaucoma surgery might induce keratometric changes depending on sutures and their tension, and further changes may occur if those sutures are removed or lysed, negating any benefit from the toric implant.

In some cases especially those with angle closure glaucoma there is poor pupillary dilation and a bad quality of pupillary function. This is particularly true of patients who have received pilocarpine for an extended period and in those who have undergone a laser iridotomy.

### Any Effect on Imaging?

Multifocal lenses affect the monitoring of patients. A recent study revealed that multifocal IOLs cause wavy artifacts on optical coherence tomography images.^34^

### Any Effect on Visual Field Assessment?

Reduced contrast sensitivity with multifocal IOLs may depress raw values, gray scale and mean deviation values. Further, increased glare may reduce the sensitivity. Frequency doubling technology perimetry is less likely to be affected as the target size is larger and hence less dependent on patients' refraction.

Patients with a diffractive MFIOL have been shown to have a clinically significant reduction of the visual sensitivity as assessed with SAP size III and size V. The reduction seems to be related to the multifocal design of the IOL rather than to pseudophakia. The reduction interferes with the assessment of common eye diseases, such as glaucoma and comes on top of the decline of visual sensitivity due to normal aging or age-related eye diseases, thus potentially accelerating visual impairment.^35^

### Does Pupil Size Alter the Decision to Put a Premium IOL?

In some patients long-term medical therapy induces pupil rigidity, and in these cases, it is better to avoid multifocal IOLs if pupil diameter is less than 3.5 mm. However, diffractive multifocal IOLs, which are not pupil-size dependent, can be considered in patients with miosed pupils.

Irregular pupil shape in eyes which have had an angle closure attack may increase the photopic symptoms.

### Which Glaucoma Patients are Potential Candidates for a Multifocal IOL?^36^

Glaucoma suspects and ocular hypertensive patients with no disk or visual field damage who have been stable.

- Glaucoma patients with early or mild visual field damage that has been controlled and stable.
- Patients with a level of glaucoma in the fellow eye that is similar, and not severe, advanced or progressive.

Due to paucity of scientific evidence in the form of large trials on the impact of MFIOL's in glaucoma, decisions regarding the implantation of a multifocal IOL in a glaucoma patient should be tailored as per the patients' motivation and the rate of progression of glaucoma. Thus, while it is not wise to implant a multifocal IOL in a patient with advanced disease the benefits of multifocality should not be denied to a patient who is motivated for the same and has a controlled stable disease.^37^
